# Novel method for retrieving a migrated plastic stent using an 11.5-Fr pusher sheath: The stent encapsulation method

**DOI:** 10.1055/a-2239-3065

**Published:** 2024-02-07

**Authors:** Fumitaka Niiya, Masataka Yamawaki, Jun Noda, Tetsushi Azami, Yuichi Takano, Fumiya Nishimoto, Masatsugu Nagahama

**Affiliations:** 1 Internal Medicine, Division of Gastroenterology, Showa University Fujigaoka Hospital, Yokohama, Japan

Plastic stent placement using endoscopic retrograde cholangiopancreatography (ERCP) is a widely used biliary drainage technique 1 . Migration of the plastic stent is a major adverse effect. Retrieval grasping techniques using baskets, snares, forceps, and balloon catheters have been reported 2 ; however, these techniques are not always successful because of biliary stenosis and tight anchoring of plastic stent flaps 3 . Herein, we introduce a novel method for retrieving a migrated plastic stent using an 11.5-Fr pusher sheath.

A 58-year-old woman with a history of multiple endoscopic treatments, including placement of a metallic stent and a plastic stent inside the metallic stent, for distal biliary obstruction caused by malignant lymphoma, was admitted to our institution because of cholangitis ( Fig. 1 ). Computed tomography revealed a dilated intrahepatic bile duct, which was suspected to be related to stent occlusion. We performed endoscopic retrograde cholangiopancreatography (ERCP) and attempted to retrieve the plastic stent using forceps, a snare, and a balloon catheter; however, these attempts failed because of the tight anchoring of a flap of the plastic stent to the metallic stent and the migration of the plastic stent into the bile duct.

**Fig. 1:**
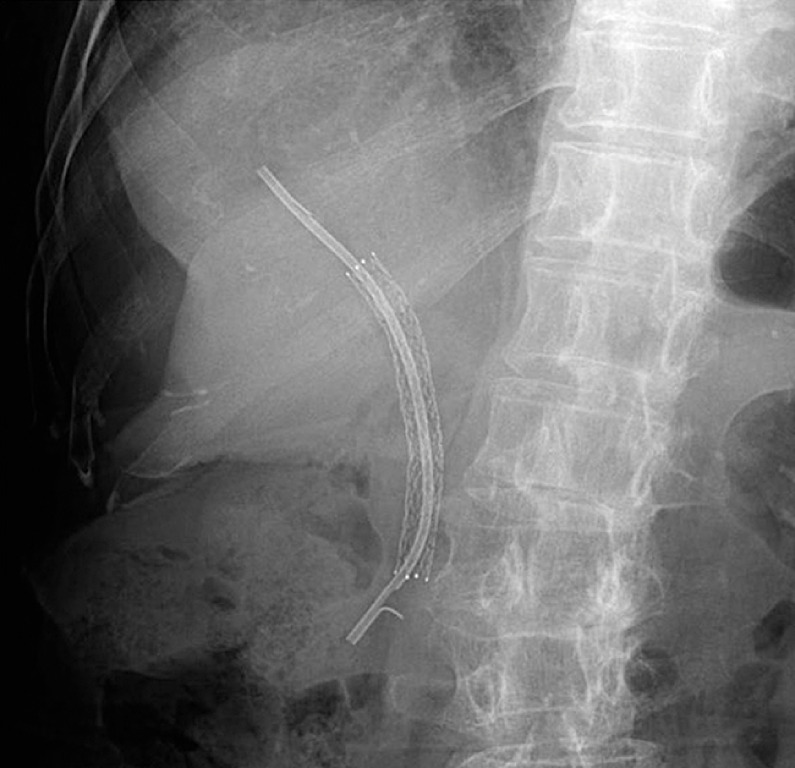
X-ray of 58-year-old woman showing possibly occluded plastic stent coaxial within a metallic stent, placed for distal biliary obstruction caused by malignant lymphoma.

Therefore, we decided to try a stent encapsulation method ( Video 1 ). We passed a 0.025-inch guidewire through the plastic stent and advanced an 11.5-Fr pusher sheath (Oasis; Cook Medical, Bloomington, Indiana, USA) over the guidewire. Forceps (E634045; Olympus Medical Systems, Tokyo, Japan) were then inserted through the pusher sheath ( Fig. 2 ). The end of the plastic stent was then grasped with the forceps ( Fig. 3 ). The 11.5-Fr pusher sheath device was advanced into the bile duct over the forceps encasing the entire length of the plastic stent ( Fig. 4 ), including the flap. The plastic stent was then removed successfully through the pusher sheath. Finally, another metallic stent was deployed inside the previously placed metallic stent ( Fig. 5 ).

Successful removal of a migrated and tightly stuck biliary plastic stent using a novel stent encapsulation method.Video 1

**Fig. 2:**
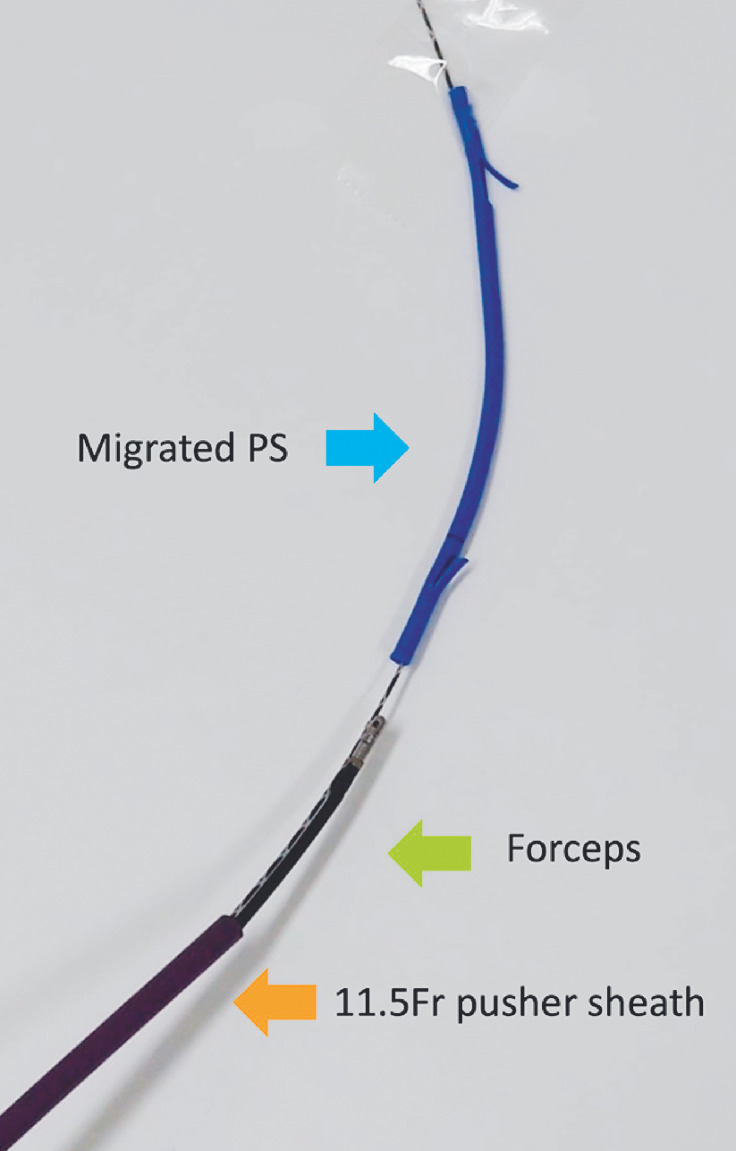
Forceps are passed through an 11.5-Fr pusher sheath on the guidewire.

**Fig. 3:**
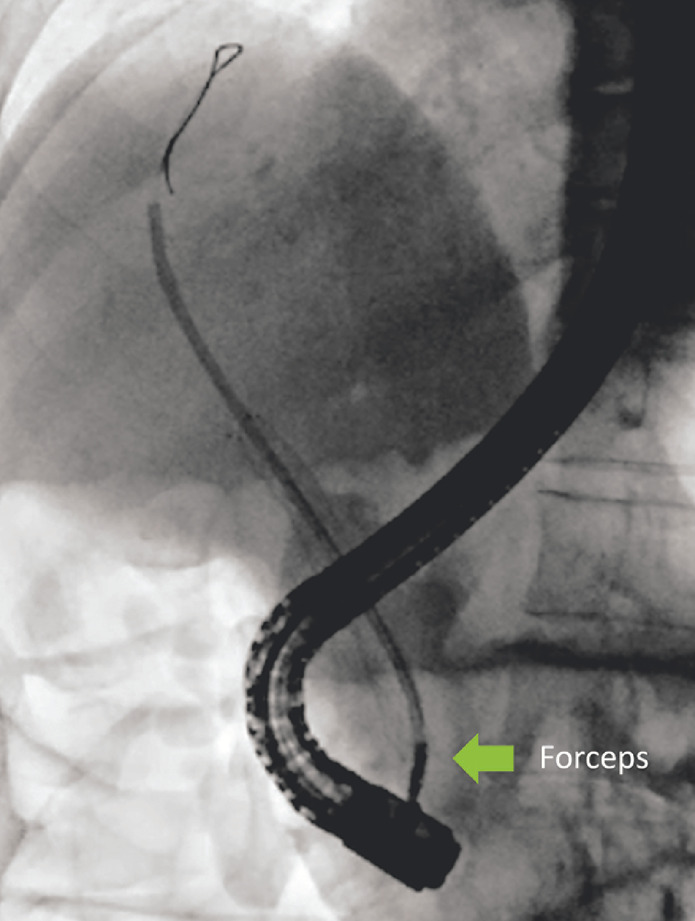
The forceps are used to grasp the end of the migrated plastic stent.

**Fig. 4:**
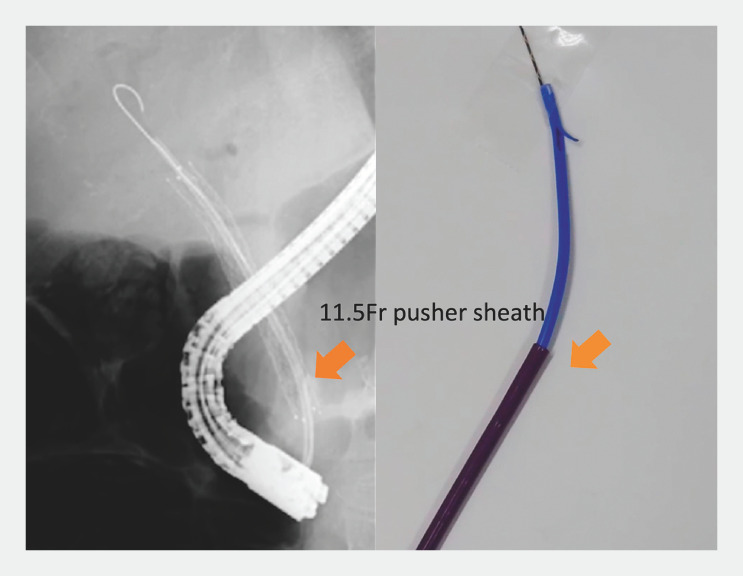
The 11.5-Fr pusher sheath is advanced over the forceps to encase the entire length of the migrated stent.

**Fig. 5:**
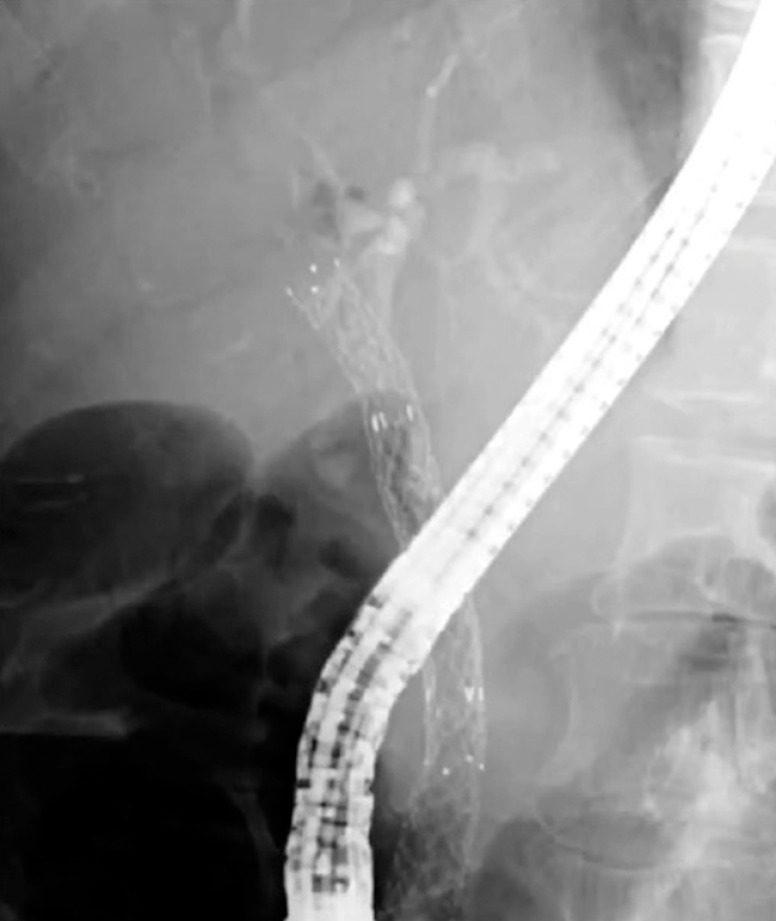
Another metallic stent is deployed inside the previously placed metallic stent.

This novel method can be useful for removing a migrated plastic stent after advancement of a sheath device beyond a stricture.

Endoscopy_UCTN_Code_TTT_1AR_2AZ

## Notes

Endoscopy E-Videos https://eref.thieme.de/e-videos
*E-Videos* is an open access online section of the journal *Endoscopy* , reporting on interesting cases and new techniques in gastroenterological endoscopy. All papers include a high-quality video and are published with a Creative Commons CC-BY license. Endoscopy E-Videos qualify for HINARI discounts and waivers and eligibility is automatically checked during the submission process. We grant 100% waivers to articles whose corresponding authors are based in Group A countries and 50% waivers to those who are based in Group B countries as classified by Research4Life (see: https://www.research4life.org/access/eligibility/ ). This section has its own submission website at https://mc.manuscriptcentral.com/e-videos .
